# The Cognitive City Cybersecurity Readiness Index (C3RI): an evidence-based model for AI-driven urban infrastructure

**DOI:** 10.3389/fdata.2026.1949829

**Published:** 2026-10-28

**Authors:** Reem Alshareef, Mahmood Niazi, Mohammad Alshayeb, Sajjad Mahmood

**Affiliations:** 1 Department of Information and Computer Science, King Fahd University of Petroleum and Minerals, Dhahran, Saudi Arabia; 2 Department of Computer Information Systems, College of Computer Science and Information Technology, Imam Abdulrahman Bin Faisal University, Dammam, Saudi Arabia; 3 Interdisciplinary Research Center for Intelligent Secure Systems, King Fahd University of Petroleum and Minerals, Dhahran, Saudi Arabia

**Keywords:** AI security, cognitive cities, critical infrastructure protection, cybersecurity readiness, IoT security, readiness model, security assessment, smart cities

## Abstract

**Introduction:**

Cognitive cities integrate autonomous decision-making, machine learning, and Internet of Things networks to create adaptive urban environments. These technologies introduce cybersecurity challenges that existing enterprise and smart city frameworks do not adequately address. This study develops an evidence-based readiness model to assess and advance cybersecurity preparedness in cognitive cities.

**Methods:**

A three-phase methodology develops the model. Phase 1 elicits cybersecurity best practices through a focused review mapped to documented cognitive city security challenges, Phase 2 organizes the practices into knowledge areas and readiness levels, and Phase 3 evaluates the result through a panel of ten cybersecurity experts and an initial application case study in a large-scale real-world cognitive city.

**Results:**

The resulting Cognitive City Cybersecurity Readiness Index (C3RI) comprises 117 best practices organized into eight Cybersecurity Knowledge Areas and distributed across five progressive readiness levels (Foundational, Structured, Intelligent, Autonomous, and Adaptive). A Motorola-based assessment scores each practice across Approach, Deployment, and Results, and a 7.0 advancement gate governs progression between levels. In the case study, level means range from 7.04 to 7.52 and exceed the gate at every readiness level. The expert review supports the clarity, coverage, and practical applicability of the model.

## 1 Introduction

Cities worldwide face growing pressure to improve operational efficiency, sustainability, and quality of life as their populations grow (Ismagilova et al., 2019). In response, they have progressively adopted advanced information technologies and evolved through three stages, namely conventional cities with minimal digital integration, smart cities that collect and analyze data to optimize services, and cognitive cities that add autonomous learning and adaptive response (Machin et al., 2021). For example, while a conventional city relies on fixed transit schedules that require manual adjustment, a smart city dynamically reroutes based on real-time traffic data, and a cognitive city anticipates future demand by analyzing weather, events, and behavioral patterns to optimize resources before problems arise.

Cognitive cities advance beyond smart city models by integrating artificial intelligence (AI), machine learning (ML), edge computing, and extensive Internet of Things (IoT) networks (Almusaed et al., 2025). These systems sense urban patterns, learn from data, and adapt without constant human intervention (Balfagih, 2025; Finger and Portmann, 2016). What distinguishes cognitive cities is their integration of IoT ecosystems, big data streams, and AI through collaborative sensor-actuator networks and human-machine interfaces (Almusaed et al., 2025), enabling autonomous learning and behavioral adaptation that smart cities cannot achieve. They can also perceive, interpret, and proactively respond to dynamic changes by drawing on historical data and patterns (Alraddadi et al., 2025b). A further distinguishing characteristic is that citizens function as active “human sensors,” contributing real-time observations that enrich system intelligence (Nayar and Raj, 2023; Mostashari et al., 2011). This shift from reactive smart cities to proactive cognitive cities represents a move toward more resilient and human-centered urban development (Alraddadi et al., 2025b).

However, the same technologies that enable cognitive cities also amplify cybersecurity risks in ways that differ fundamentally from those in traditional smart city environments. In isolated critical infrastructure such as a single traffic system or power grid, security breaches tend to cause more contained damage because isolation limits the propagation path. In cognitive cities, AI decision-making is deeply interconnected across urban services, and interconnection creates propagation paths by design. A single compromised IoT sensor feeding false data into the traffic management system could simultaneously degrade emergency response routing, public transit scheduling, and traffic signal optimization, creating a cascading failure that is difficult to contain or isolate. The deep reliance on artificial intelligence introduces threat vectors that are marginal in conventional infrastructure but pervasive in cognitive cities, namely adversarial machine learning attacks that poison training data, model extraction attacks that steal proprietary models, and emerging autonomous malware that adapts in real time to defensive countermeasures (Alraddadi et al., 2025a). Cognitive cities are distinctively exposed to these vectors because AI decision-making is pervasive and interconnected across their services. The increased attack surface creates qualitatively different security challenges that call for tailored readiness assessments, a need existing frameworks do not meet (Machin et al., 2021).

Although cities are shifting their focus toward cognitive systems, the cybersecurity literature has concentrated on smart cities. Few frameworks target the complexities specific to cognitive cities, particularly adversarial ML threats and the security of autonomous decision-making at the urban scale. Frameworks designed for the urban scale treat security as a secondary concern. Smart city maturity models measure dimensions such as resilience, sustainability, and connectivity (Afonso et al., 2015; Suliman et al., 2021), and they position security and privacy as sub-criteria under ICT or governance rather than as standalone dimensions with systematic progression pathways. ISO 37122 similarly emphasizes performance indicators for smart, sustainable cities rather than security readiness progression (ISO 37122, 2019). Moreover, frameworks designed for security are not formalized for the urban scale. The NIST Cybersecurity Framework 2.0 (NIST, 2024), the NIST AI Risk Management Framework (NIST, 2023), and industrial and IoT security standards provide comprehensive control catalogs, risk functions, and architectural guidance but do not define a multi-level, citywide cybersecurity readiness scale. Section 2.2 analyzes these standards and frameworks in detail.

The verification search reported in Section 2 retrieved no evidence-based, empirically evaluated security-specific readiness model for cognitive cities. Our prior multivocal literature review and expert prioritization study (Alshareef et al., 2026) identified and ranked six categories of cybersecurity challenges specific to cognitive cities, establishing the problem space this work addresses. Building on that foundation, this study develops a tailored readiness model that integrates technical, organizational, governance, and policy dimensions, maps evidence-based practices to progressive readiness levels, and provides practical assessment methods to guide security investments and resilience development. Without such a model, cities assemble ad-hoc combinations of separate cybersecurity frameworks, leaving critical dimensions overlooked.

This research addresses three research questions:

**RQ1:** what are the best practices for assessing and improving cybersecurity readiness in cognitive cities?
**RQ2:** what knowledge areas are essential for cognitive city cybersecurity readiness?
**RQ3:** how can a Cognitive City Cybersecurity Readiness Index (C3RI) model be developed and applied?

This paper makes five contributions:

- **C3RI model:** to our knowledge, the first evidence-based and empirically evaluated security-specific readiness model for cognitive cities, with five readiness levels integrating technical, organizational, governance, and policy dimensions.
- **Evidence-based practice library:** 117 best practices mapped to eight Cybersecurity Knowledge Areas (CKAs) and five readiness levels, traceable to international standards or peer-reviewed sources.
- **Motorola-based assessment approach:** a structured assessment methodology, built on the established Motorola scoring method (Daskalantonakis, 1994), that scores each practice across approach, deployment, and results, with a defined advancement gate that governs progression between readiness levels.
- **Practical assessment tool:** a tool that automates Motorola calculations and provides a dashboard for advancement-gate status.
- **Expert review and initial application case:** empirical evaluation through a review with 10 cybersecurity specialists and an initial application case study of a large-scale national cognitive city initiative (City X), including knowledge-area and practice-level gap diagnostics that illustrate the model's use as an investment-prioritization instrument.

Figure 1 presents the C3RI at a glance, namely the five readiness levels, the eight Cybersecurity Knowledge Areas that organize the 117 practices, and the assessment method. Section 4 details each element.

**Figure 1:**
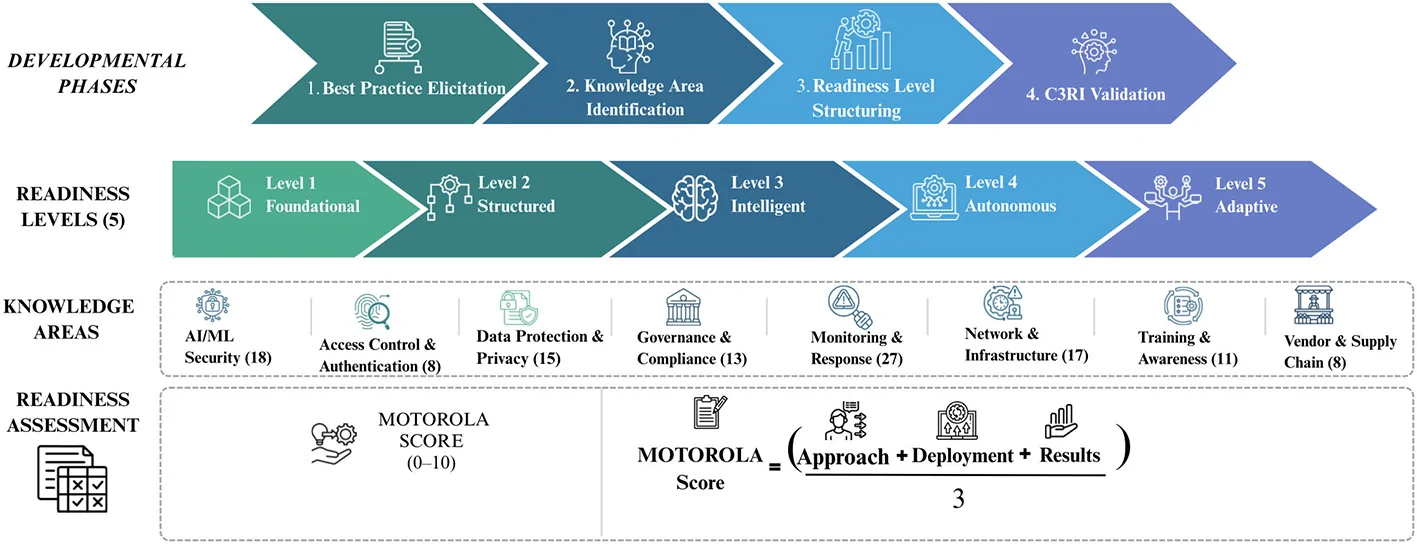
The C3RI model overview.

The remainder of this paper is organized as follows. Section 2 reviews related work. Section 3 describes the three-phase research methodology. Section 4 presents the C3RI architecture, readiness levels, knowledge areas, and best practices. Section 5 presents C3RI validation results. Section 6 discusses the results. Section 7 examines limitations, and Section 8 concludes with directions for future research.

## 2 Related work

Maturity and readiness models provide structured mechanisms for assessing and advancing organizational capabilities in complex technological or operational domains. These models organize progression through levels, enabling gap analysis and targeted improvement. Although such models are well-established across several fields, none fully captures the specific characteristics of cognitive cities. To verify whether a security-specific readiness model already exists for cognitive cities, we initially ran a search in Scopus, IEEE Xplore, the ACM Digital Library, and Web of Science on titles, abstracts, and keywords in February 2026, with no date restriction, using the string (“cognitive city” OR “cognitive cities” OR “smart city” OR “smart cities” OR “intelligent cities”) AND (“cybersecurity” OR “cyber security” OR “information security”) AND (“readiness model” OR “maturity model” OR “readiness index” OR “maturity levels”). A record was retained if it proposed a model whose primary object of assessment is security, organized that assessment into ordered levels or stages, and targeted smart, cognitive, or AI-driven cities. Records that treated security as one sub-dimension of a general smart-city maturity model, that assessed threats or risks without a level structure, or that addressed a single sector (for example, transport or energy) were excluded. The closest match retrieved by this search is the work of Al Shidhani (2019), which proposes ordered cyber-defense maturity levels for smart cities together with threat models associated with each level. That work, however, is a conceptual proposal rather than an assessment model and targets smart rather than cognitive cities. As a result, no retrieved source constitutes a validated, security-specific readiness assessment model for cognitive or AI-driven cities. The review below first examines domain-specific readiness and maturity models, then analyzes the international standards landscape for AI, IoT, and critical infrastructure security, and locates the assessment gap that C3RI addresses.

### 2.1 Cybersecurity readiness and maturity models

Wu (2004) developed an early framework for technology adoption readiness, proposing a model for Web services adoption. The model comprises four constructs, namely innovation awareness readiness, innovation-needs fit readiness, technological skills readiness, and financial resources readiness. Although Wu acknowledged the model required empirical validation, this work established a foundation for domain-specific readiness assessment. Building on this foundation, a series of security readiness models has emerged, focusing on integrating security practices into the software development lifecycle (SDLC) phases. These models provide methodological foundations relevant to the C3RI framework, particularly in their use of maturity-based structures and evidence-based practice mapping. Mufti et al. (2018) introduced a Readiness Model for Security Requirements Engineering (SRERM). Through systematic mapping of 104 primary studies, the authors synthesized evidence into 12 security requirements categories and 76 best practices organized across five maturity levels. Four iterative case studies in organizational settings refined the model and provided evidence of its applicability for assessing readiness to incorporate security during the requirements phase. Similarly, Al-Matouq et al. (2020) developed a Secure Software Design Maturity Model (SSDMM) through a multivocal literature review of 38 primary studies. Evidence was synthesized into 8 knowledge areas and 65 best practices, structured using the Capability Maturity Model Integration (CMMI) v2.0 as a foundational framework. Case-study evaluation in organizational settings provided preliminary evidence of the SSDMM's applicability for assessing secure-design practices and guiding the early integration of security principles. Humayun et al. (2023) proposed the Secure Software Coding Readiness Model (SSCRM). The model organizes coding-related security challenges and evidence-based best practices across five maturity levels. They also map specific secure coding challenges to corresponding best practices at each level, enabling phased implementation and assessment of an organization's capacity to produce secure code. Validation through three case studies demonstrated the model's practical utility in determining organizational secure coding readiness. Extending readiness assessment to the software supply chain, Alnaseef et al. (2023) developed the Readiness Model for Secure Software Acquisition (RMSSA). This model addresses the critical challenge of assessing third-party supplier capabilities through a systematic literature review that identified best practices across six core security knowledge areas (KAs), namely confidentiality, integrity, availability, authorization, authentication, and accountability. Validated through theoretical evaluation and three organizational case studies, RMSSA enables software-acquiring organizations to evaluate supplier readiness to provide secure software.

Other models look beyond technical processes to people and organizations. Abiodun et al. (2025) presented a cybersecurity readiness model based on human factors. Through a multivocal literature review of 120 primary studies, the authors identified human-centric barriers (trust deficits, ignorance, and lack of technological knowledge) and success factors (knowledge acquisition, proactive awareness, cognitive ability) that influence organizational cybersecurity preparedness. The identified barriers and success factors were mapped to the CyBOK (Cyber Security Body of Knowledge) framework, which gives structured guidance for the socio-technical dimensions of cybersecurity readiness. This work emphasizes that technical controls alone are insufficient without addressing behavioral, cultural, and awareness-related factors. Al Zeibak et al. (2025) proposed a cybersecurity maturity model tailored to digitally transformed organizations. Using two multivocal literature reviews of 165 studies, they identified seven cybersecurity capability areas and 22 practice areas. This work reinforces the need to align cybersecurity assessment with the characteristics of digital transformation rather than relying exclusively on generic cybersecurity models.

Closest to the present work, Al Shidhani (2019) examines the technical and non-technical cybersecurity challenges facing smart cities and outlines design requirements for a secure smart city spanning the technical, operational, social, safety, legal, and usability domains. The author proposes cyber-defense maturity levels for smart cities in which each maturity level is associated with the threat models it is expected to withstand. The C3RI differs from this work in scope and structure. In scope, Al Shidhani (2019) addresses smart cities, whereas the C3RI targets cognitive cities and the adversarial machine learning, AI-agent authentication, and autonomous-response threats that AI-driven urban operation introduces. In structure, the maturity levels of Al Shidhani (2019) are presented as conceptual level descriptions supported by design requirements, without a practice library, a scoring instrument, or a defined advancement criterion. By contrast, the C3RI operationalizes each of its five levels through an assessable practice set, Motorola scoring of Approach, Deployment, and Results, and an explicit advancement gate.

The C3RI differs from SRERM, SSDMM, SSCRM, and RMSSA in several respects. The previously discussed models assess a software-producing or software-acquiring organization at one phase of the development lifecycle (requirements, design, coding, or acquisition), whereas the C3RI assesses a city-scale cyber-physical ecosystem in which AI-mediated services are interdependent. Additionally, the lifecycle models and the digital-transformation model of Al Zeibak et al. (2025) order levels by the institutionalization of processes, whereas the C3RI levels are ordered by the degree of sophistication that the security capability itself requires, from manual controls through human-in-the-loop analytics to human-on-the-loop autonomy and self-evolving defenses. Finally, the threat scope differs, as the C3RI practice library covers advanced practices involving adversarial machine learning, AI-agent authentication, resource-constrained IoT security, and autonomous response, which are absent from these models.

Table 1 summarizes these readiness and maturity models. Together they show that a credible readiness model rests on literature-grounded practice identification, expert evaluation, and a multi-level progressive structure, and the C3RI adopts the same methodological approach in a domain these models do not target.

**Table 1:** Summary of maturity and readiness models.

Reference	Year	Purpose	Readiness/ maturity levels	Empirical validation	# of Best Practices
Mufti et al. (2018)	2018	Security requirements engineering	5	4 case studies	76
Al-Matouq et al. (2020)	2020	Secure software design	4	2 case studies	65
Humayun et al. (2023)	2023	Secure software coding	5	3 case studies	24
Alnaseef et al. (2023)	2023	Secure software acquisition	Used KAs instead of levels	3 case studies + Academic evaluation with 3 professors	104
Abiodun et al. (2025)	2025	Human factors cybersecurity	5	2 case studies	85
Al Zeibak et al. (2025)	2025	Digital transformation cybersecurity	5	5 case studies + expert judgments	179
**C3RI (this study)**	2026	Cognitive city cybersecurity	5	Expert panel (*n* = 10) + 1 case study	117

Bold indicates the model developed in the present study.

### 2.2 Existing standards and frameworks

To assess whether international security standards cover cognitive cities, we group the relevant standards and frameworks into five families and assess each against the requirements of city-scale readiness assessment as follows.

- **Enterprise security management:** ISO/IEC 27001 (ISO/IEC 27001, 2022) and ISO/IEC 27002 (ISO/IEC 27002, 2022) define information security management system requirements and a reference catalog of controls. NIST CSF 2.0 (NIST, 2024) organizes security outcomes into six functions (Govern, Identify, Protect, Detect, Respond, and Recover) with four implementation tiers as descriptors of risk-governance rigor rather than a readiness scale. These instruments are deliberately sector-neutral and organization-scoped. Neither prescribes a progression pathway toward autonomous, AI-mediated operations.
- **AI governance and AI threat knowledge:** the NIST AI Risk Management Framework (NIST, 2023) and ISO/IEC 42001 (ISO/IEC 42001, 2023) codify AI risk management processes. MITRE ATLAS (MITRE Corporation, 2025) catalogs adversarial tactics and techniques observed against deployed machine learning systems. These artifacts are indispensable inputs to cognitive city security, and several C3RI practices at Levels 3–5 operationalize them. They remain risk-management process frameworks and threat knowledge bases rather than readiness scales, and they are not calibrated for interdependent urban service ecosystems.
- **Industrial and IoT security:** ETSI EN 303 645 (ETSI EN 303 645, 2020) and NISTIR 8259 (Fagan et al., 2020) establish baseline security requirements for consumer IoT devices and their manufacturers. ENISA provides baseline security recommendations for IoT in critical information infrastructures (ENISA, 2017). These standards are device and system scoped, not suitable for cognitive cities that are operating large numbers of interdependent systems and services.
- **Supply chain security:** NIST SP 800-161 (NIST, 2022) codifies cybersecurity supply chain risk management practices, which the C3RI draws on in its Vendor and Supply Chain knowledge area, but at enterprise granularity rather than city scale.
- **Urban indicators and maturity models:** ISO 37122 (ISO 37122, 2019) specifies performance indicators for smart cities, and smart city maturity models (Afonso et al., 2015; Suliman et al., 2021) measure urban smartness across dimensions such as resilience, sustainability, and connectivity. In both, security appears as a sub-criterion rather than as a main, progressively assessed capability. National cyber authorities have begun to close this gap from the practice side. Joint guidance from CISA, NSA, and FBI with international partners sets out cybersecurity best practices for smart cities (Cybersecurity and Infrastructure Security Agency et al., 2023), and CERT-In publishes security guidelines for smart city infrastructure (Indian Computer Emergency Response Team, 2025). Both are control recommendations rather than readiness scales, and neither treats AI-specific threats systematically.

The current standards are catalogs and process frameworks and do not specify which capabilities a city must have in place before it can safely operate AI-assisted, autonomous, or self-adapting defenses. Conformity with a standard is assessed as present or absent (or certified or not), whereas the Motorola instrument scores Approach, Deployment, and Results separately on a 0–10 scale for every practice, capturing commitment, execution, and demonstrated outcome. Thus, partially deployed controls and controls without demonstrated results are distinguished and can be prioritized. Table 2 consolidates this analysis; none combines AI/ML-specific threat coverage, city-scale applicability, and progressive readiness levels with a defined assessment instrument. The C3RI is therefore designed as a complementary overlay. It does not replace these standards but operationalizes them for cognitive city cybersecurity readiness assessment, addressing the absence that Vitunskaite et al. (2019) identify, namely that conventional cybersecurity standards and frameworks do not directly address the security dimension of smart cities and that this absence threatens the functionality of urban infrastructure.

**Table 2:** Comparative positioning of C3RI against established frameworks and standards.

Framework/model	Primary scope	AI/ML	City	Levels	Readiness
NIST CSF 2.0 (NIST, 2024)	Enterprise and critical infrastructure risk management	Partial	Partial	4 tiers^a^	No
NIST AI RMF 1.0 (NIST, 2023)	AI risk management	Yes	No	No	Partial
ISO/IEC 27001/27002 (ISO/IEC 27001, 2022; ISO/IEC 27002, 2022)	Information security management	No	No	No	No
ISO/IEC 42001 (ISO/IEC 42001, 2023)	AI management systems	Yes	No	No	No
ETSI EN 303 645; NISTIR 8259 (ETSI EN 303 645, 2020; Fagan et al., 2020)	IoT device baseline security	No	No	No	No
Smart city maturity models (Afonso et al., 2015; Suliman et al., 2021)	Urban smartness (security as sub-criterion)	No	Yes	Yes	Partial
ISO 37122 (ISO 37122, 2019)	Smart city performance indicators	No	Yes	No	No
Cyber-defense maturity levels (Al Shidhani, 2019)	Smart city cyber defense (conceptual, with per-level threat models)	No	Yes	Yes	No
**C3RI (this study)**	Cognitive city cybersecurity	**Yes**	**Yes**	**5 levels**	**Yes**

^a^NIST characterizes CSF tiers as descriptors of risk-governance rigor, not a maturity scale. Bold indicates the model developed in the present study.

## 3 Methodology

The C3RI was developed through a systematic, three-phase methodology, shown in Figure 2. Each phase builds on the one before it. Phase 1 elicits cybersecurity best practices from the literature, Phase 2 organizes them into the readiness model, and Phase 3 evaluates the result.

**Figure 2:**
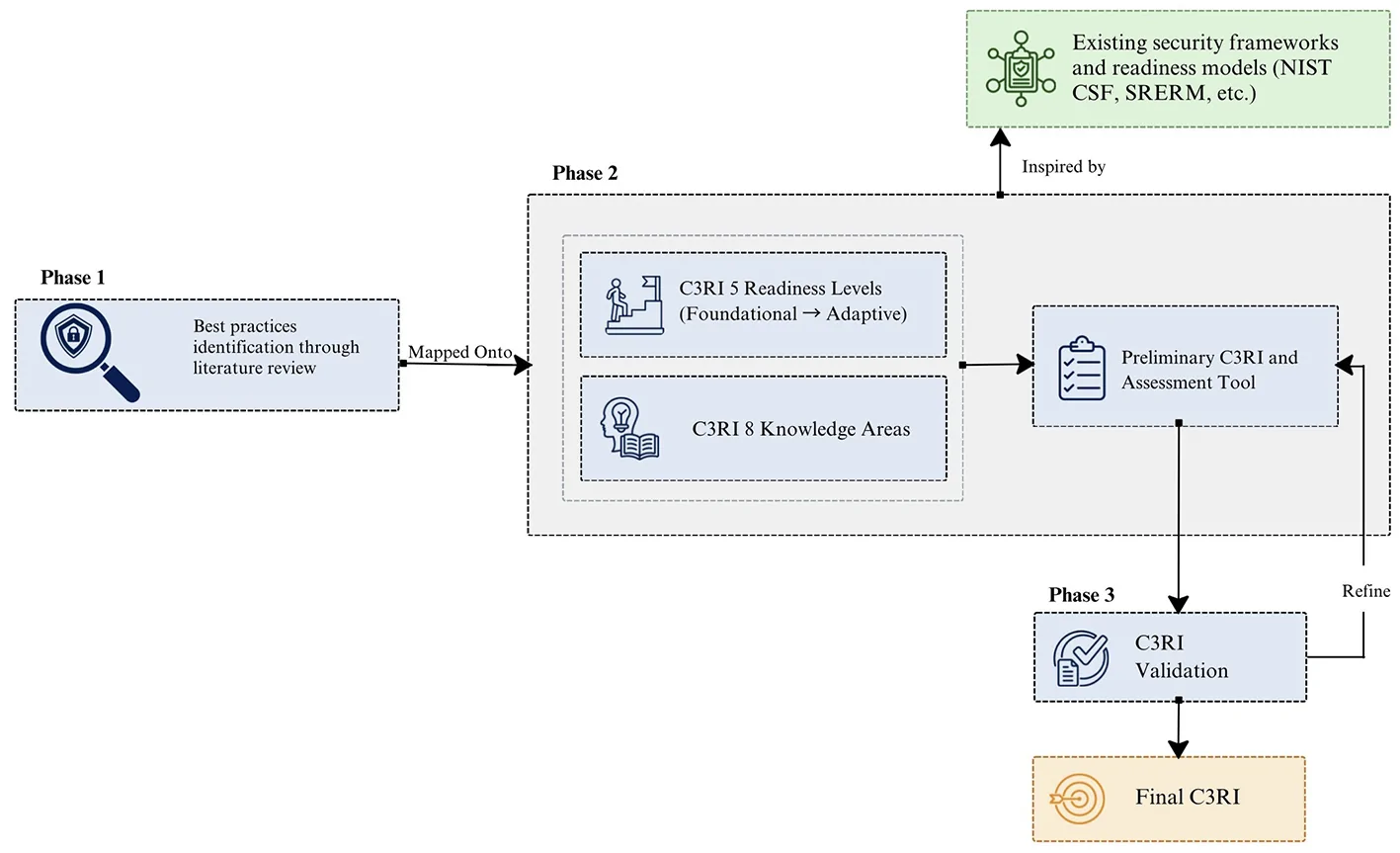
Research methodology.

### 3.1 Phase 1: focused literature review for best practice elicitation

We conducted a formal, focused literature review to identify evidence-based cybersecurity best practices, standards, and controls relevant to cognitive city environments. The review followed a challenge-driven design rather than a systematic-review pipeline over bibliographic records. Its starting point was the cognitive-city cybersecurity challenge space established through our prior multivocal literature review and expert prioritization study (Alshareef et al., 2026), and for each identified challenge we searched for documented solutions, controls, and practices capable of addressing it. The purpose of Phase 1 was therefore to populate a practice library whose coverage is defined by this previously established threat space. The review drew on three families of sources, namely peer-reviewed literature on cognitive and smart city security; international standards and frameworks, including NIST CSF 2.0 (NIST, 2024), the NIST AI Risk Management Framework (NIST, 2023), the ISO/IEC 27000 series (ISO/IEC 27001, 2022; ISO/IEC 27002, 2022; ISO/IEC 27035-1, 2023), and ISO/IEC 42001 (ISO/IEC 42001, 2023); and domain-specific security guidance for AI/ML, IoT, and cyber-physical systems, including the MITRE ATLAS adversarial threat knowledge base (MITRE Corporation, 2025). Candidate practices were elicited from these sources in two complementary ways. Standards and guidance documents were examined for controls and recommendations applicable to the challenge space. For the peer-reviewed literature, challenge-specific searches were conducted in Scopus, IEEE Xplore, the ACM Digital Library, and Web of Science, combining terms representing each challenge with solution-oriented terms such as “defense,” “mitigation,” and “best practices.” For example, [“adversarial machine learning in smart cities” AND (“defense” OR “mitigation”)], and relevant results were examined. The sources from which each retained practice was elicited were recorded and are available in the companion repository. A candidate practice was retained only when it satisfied three criteria, namely relevance, assessability, and evidence. Each was operationalized as a binary screening question.

- *Relevance*: can the practice be traced to at least one of the cognitive-city cybersecurity challenges identified in Alshareef et al. (2026)?
- *Assessability*: can an assessor identify evidence of commitment, deployment breadth, and outcome for the practice, allowing it to be meaningfully scored across the Approach, Deployment, and Results dimensions?
- *Evidence*: can at least one standard clause, peer-reviewed study, or published guidance document be cited in support of the practice?

Screening was performed by the first author and independently checked by a second author against the same questions, with disagreements resolved through discussion. Overlapping formulations of the same control from multiple sources were consolidated into a single practice statement. The resulting practice set was subsequently evaluated through the expert review described in Section 5.

The relevance criterion also defined the conceptual boundary of the practice library. Our prior study organized the identified challenges through thematic analysis into six categories comprising challenge groups and key issues, each traceable to the sources reporting it (Alshareef et al., 2026), and a practice entered the library only as a mitigation of a documented key issue. Relevance was further applied with a security-objective reading, the practice had to protect the confidentiality, integrity, availability, authenticity, or accountability of cognitive-city assets, or serve as an enabler without which relevant security controls could not operate. For instance, explainability practices (CC-BP-1, CC-BP-6) address AI/ML algorithmic opacity, which prevents the validation and auditing of AI decisions affecting security. The training-data practice (CC-BP-4) addresses the integrity of information used in AI, the surface exploited by data poisoning. The resource-management, energy, and wireless practices address the computational, energy, and communication constraints of city devices, which can limit the deployment of robust security controls. The challenge group mapping is available in the companion repository.

### 3.2 Phase 2: C3RI development

The practices were organized into Cybersecurity Knowledge Areas and placed within a five-level readiness model, as detailed below.

#### 3.2.1 Knowledge area identification

The elicited practices were organized using thematic analysis (Braun and Clarke, 2006). Two researchers independently assigned descriptive codes to each practice based on its security domain and operational focus, then grouped related codes iteratively until coherent, higher-level themes emerged. Codes were merged or divided to keep each theme internally consistent, and disagreements were resolved through discussion. This process yielded eight primary CKAs, which answer RQ2 by naming the knowledge domains essential for cognitive city cybersecurity readiness. Each practice was assigned to exactly one primary CKA on the basis of the descriptive codes from the thematic analysis, which captured each practice's security domain and operational focus. Where a practice could be coded to more than one area, the tie was resolved toward the area whose security objective the practice primarily serves, that is, the domain that would be directly compromised if the practice were absent. For example, machine-learning anomaly detection over urban datasets (CC-BP-69) uses AI but exists to protect the integrity of AI inputs, so its primary CKA is AI/ML Security rather than Monitoring and Response. We considered a multi-label scheme and did not adopt it as multi-label membership would double-count practices in the knowledge-area diagnostics of Section 5 and make the eight area scores non-additive. To confirm that this eight-area scheme could be applied consistently, the reliability of the practice-to-CKA mapping was assessed using an automated coding procedure. Four independent large language model coders, two from each of two model families, were used as a supplementary automated consistency check on the assignment of all practices. The procedure and its agreement results are detailed in Section 4.6.

#### 3.2.2 Readiness level structuring

The five readiness levels form a cumulative staged scale, consistent with CMMI-based readiness models such as those presented by Mufti et al. (2018) and Humayun et al. (2023). An organization attains a level when the mean score for that level meets the advancement threshold and all preceding levels have been attained. The advancement gate described in Section 4.5.2 implements this rule using the Motorola assessment instrument (Daskalantonakis, 1994).

The ordering construct is the class of operational capability a practice presupposes, that is, the degree to which its operation delegates security decision-making to machines. Level 1 practices need no deployed mechanism and rest on governance, policy, or manual procedure. Level 2 practices use deployed, rule-based procedures. Level 3 practices use machine learning or analytics whose outputs a human acts on. Level 4 practices act autonomously, with humans reviewing actions after execution. Level 5 practices act independently and modify their own models, rules, or thresholds from experience. This ordering reflects how a practice operates, not how novel its technology is; post-quantum cryptography, for example, is novel technology with a static, rule-based operation, so it sits at Level 2.

To make the assignment reproducible, each practice was placed at the level whose capability class matches its operation, using the questions in Table 3. For instance, adversarial robustness testing at Level 2 (CC-BP-37) is a systematic, human-run testing process, whereas the autonomous red-team practice at Level 5 (CC-BP-117) continuously evolves its own attack simulations from operational experience. To assess the reliability of the assignment, two experienced cybersecurity researchers classified all 117 practices independently using the criteria in Table 3. They agreed on 104 practices (88.9% observed agreement). Cohen's κ was 0.85 (95% bootstrap CI 0.77–0.92, 10,000 item-level resamples), and the linearly weighted κ, which credits partial agreement between adjacent levels, was 0.92 (95% CI 0.87–0.96); both indicate almost perfect agreement (Landis and Koch, 1977). Twelve of the 13 disagreements involved adjacent levels, eight of them practices placed at Level 3 by one researcher that the other judged implementable through the deterministic rules of Level 2. The disagreements were resolved by a third researcher through discussion until consensus was reached.

**Table 3:** Level-assignment rule.

Level	Ordered question
L1 Foundational	Q1. Can the practice be established through governance, policy, planning, or manual procedure alone?
L2 Structured	Q2. Does the practice depend on a deployed mechanism or a systematic, rule-based process that does not learn from data?
L3 Intelligent	Q3. Does the practice depend on machine learning or analytics to produce its output, with a human deciding the resulting action?
L4 Autonomous	Q4. Does the practice execute its action autonomously, without a human decision at the point of execution?
L5 Adaptive	Q5. Does the practice modify its own models, rules, or thresholds from operational experience in order to function?

The questions are applied in ascending order, and a practice is assigned to the level of the first question it satisfies.

### 3.3 Phase 3: C3RI evaluation

The model first underwent iterative internal validation by academic cybersecurity researchers. It was then evaluated externally through expert review by ten cybersecurity experts selected through purposive sampling to ensure domain relevance, and an initial application case study in a real-world cognitive city context (City X). The validation design follows established guidance for case study research in empirical software and security engineering (Runeson and Höst, 2009) and mirrors the dual-method rationale of CYSFAM (Yigit Ozkan et al., 2021), in which the authors specify that neither a case study nor an expert evaluation alone is sufficient to surface all model deficiencies. The experts assessed the model for completeness, clarity, and practical applicability in guiding cybersecurity readiness improvement in cognitive city environments. The nine-item evaluation instrument was adapted from the instruments used to validate prior readiness models (Mufti et al., 2018; Humayun et al., 2023; AlGhamdi et al., 2024), covering clarity, coverage, structure, and practical applicability; Section 5 reports the full instrument and response distributions. This phase addresses RQ3 by providing expert evaluation of the model and an initial application in a real-world cognitive city.

## 4 Cognitive City Cybersecurity Readiness Index (C3RI) Model

The three-phase methodology described in Section 3 produced a final set of 117 evidence-based best practices grounded in peer-reviewed literature and international standards, which answer RQ1. Each practice was selected for its direct relevance to cognitive city cybersecurity, not as a generically derived control. The design of the model was informed by established readiness models in the literature, including SRERM (Mufti et al., 2018), the human factors cybersecurity readiness model (Abiodun et al., 2025), and the organizational readiness model for insider attacks (AlGhamdi et al., 2024). These readiness models employ five progressive levels and have been validated through case studies conducted with organizations.

### 4.1 C3RI architecture

The C3RI is organized along four integrated components that together constitute a complete readiness assessment model.

- Five progressive readiness levels representing increasing sophistication of cybersecurity capability.
- Eight Cybersecurity Knowledge Areas that organize practices into coherent security domains.
- 117 evidence-based best practices distributed across the levels and knowledge areas.
- A Motorola-based assessment methodology that scores each practice across the Approach, Deployment, and Results dimensions.

The model builds on concepts from maturity models such as CMMI (CMMI Product Team, 2010) and C2M2 (U.S. Department of Energy, 2022), but adopts a readiness-oriented design focused on organizational preparedness to deploy and operate cognitive-city security capabilities rather than on process maturity. The two constructs differ in what their levels represent. A maturity level indicates how deeply organizational processes are institutionalized (CMMI Product Team, 2010); a C3RI level identifies the class of security capability that a city is prepared to operate, ordered according to the degree to which security decision-making is delegated to machines. A higher C3RI level therefore does not indicate greater institutionalization of the same organizational processes. Instead, it indicates assessed preparedness to operate a security capability involving a greater degree of machine-supported or autonomous decision-making. In this context, technological sophistication refers to how the capability operates rather than to the novelty of its underlying technology. The advancement gate indicates that the mean score for a capability class meets the model's preparedness threshold; it does not certify complete implementation of every practice or the institutional maturity of the surrounding processes. The present validation provides preliminary evidence concerning the applicability of this readiness structure but does not establish discriminant validity relative to a conventional cybersecurity maturity instrument.

### 4.2 Readiness levels

The C3RI defines five progressive readiness levels representing increasing sophistication of cybersecurity practices in cognitive city environments. The practice level characteristics are as follows.

- **L1 Foundational:** cities possess basic governance structures and initial control implementations. The security practices focus on foundational policies, asset inventories, and manual baseline assessments. This level establishes the minimum necessary safeguards for cognitive city infrastructure without dependence on advanced technologies.
- **L2 Structured:** cities establish organized, policy-driven security processes with layered defenses, standardized procedures, and rule-based security enhancements. Implementation becomes more systematic and consistent across operations, though execution remains primarily human-overseen.
- **L3 Intelligent:** the city's security defenses are augmented with machine learning and data analytics, enabling AI-assisted threat detection, predictive insights, and intelligent recommendations. Security teams validate and refine AI-generated insights before implementation, maintaining decision authority over critical security choices. The balance shifts toward AI, which provides machine-assisted analysis while humans retain oversight and judgment over outcomes.
- **L4 Autonomous:** systems operate independently with automated incident response, self-healing mechanisms, and pre-defined countermeasures executing without continuous human intervention. Defensive responses activate automatically when anomalies exceed predefined thresholds, with human oversight focused on critical decisions and post-incident review. The security infrastructure executes established protocols autonomously, with human-on-the-loop reviewing autonomous actions after execution and retaining decision authority over mission-critical interventions.
- **L5 Adaptive:** cities become self-optimizing, continuously learning from incidents and operational data to proactively evolve defenses and improve resilience. Systems automatically incorporate new threat intelligence, update defensive strategies based on attack patterns, and adapt controls in response to emerging threats. Defenses are continuously refined based on accumulated experience, representing the security posture characteristic of a fully realized cognitive city.

We have chosen the specific level names Foundational, Structured, Intelligent, Autonomous, and Adaptive to reflect the progression of AI-driven urban systems from basic security implementation through rule-based standardization, analytics-driven intelligence, automated autonomous response, and finally self-evolving adaptive defense.

### 4.3 Cybersecurity knowledge areas

The C3RI encompasses eight primary CKAs identified through thematic analysis of the 117 practices, as described in Section 3. These knowledge areas represent the interdependent security domains of cognitive city environments across organizational, technical, and governance considerations.

- **KA1 AI/ML security:** addresses the security and integrity of artificial intelligence and machine learning systems, encompassing model poisoning prevention, adversarial robustness, AI validation pipelines, and explainability requirements.
- **KA2 access control and authentication:** encompasses identity management, authentication mechanisms, authorization frameworks, and privilege management, extended to the machine-to-machine and AI-agent interactions that dominate cognitive infrastructure, where continuous authentication must cover AI-to-AI and human-to-AI exchanges.
- **KA3 data protection and privacy:** addresses data encryption, privacy controls, data handling procedures, and information asset protection, with particular attention to citizen-scale data collection and to the datasets on which urban AI models are trained and operated.
- **KA4 governance and compliance:** this CKA addresses the socio-technical governance demands unique to multi-stakeholder cognitive city environments. It covers security governance structures, regulatory compliance, risk management frameworks, and policy implementation.
- **KA5 monitoring and response:** covers security monitoring systems, incident detection, threat analysis, and response procedures, spanning conventional infrastructure telemetry and the behavioral monitoring of AI-driven city services whose failure modes include model drift and adversarial manipulation.
- **KA6 network and infrastructure:** covers network architecture, infrastructure security, IoT device protection, security for resource-constrained devices, and micro-segmentation.
- **KA7 training and awareness:** addresses security training programs, user awareness initiatives, workforce planning, and citizen engagement in cognitive city security.
- **KA8 vendor and supply chain:** this CKA addresses the high dependence of cognitive city infrastructure on vendor-supplied AI platforms, IoT devices, and edge computing systems whose security posture directly affects citywide cybersecurity. It addresses third-party supplier security requirements, contractual security standards, backdoor prohibitions, secure software development lifecycle requirements for vendors, and ongoing third-party risk monitoring.

### 4.4 Best practices

The practices represent established, proven approaches to address cognitive city cybersecurity challenges, grounded in academic literature, international standards, including NIST CSF (NIST, 2024) and ISO/IEC frameworks (ISO/IEC 27001, 2022; ISO/IEC 42001, 2023; ISO/IEC 27035-1, 2023), together with expert input. Each practice is assigned to one CKA and one readiness level. The concentration at Levels 1 and 2 ensures that organizations building cognitive city security from the ground up receive thorough guidance on the governance, technical controls, and organizational processes that all higher levels rely on. The smaller counts at Levels 4 and 5 reflect the specialized nature of autonomous and adaptive practices, where a small set of well-implemented practices can improve a city's cybersecurity readiness. The complete list of practices, organized by readiness level, can be found in the Supplementary material.

### 4.5 Assessment methodology

The C3RI employs a Motorola-based assessment methodology that evaluates the implementation completeness of each best practice and governs progression between readiness levels through a defined advancement gate, as detailed below.

#### 4.5.1 Motorola scoring

Each best practice is assessed across three implementation dimensions using the Motorola instrument (Daskalantonakis, 1994), which was developed at Motorola for software process assessment and has since been adopted by multiple cybersecurity and software security readiness models (Mufti et al., 2018; Humayun et al., 2023; Alnaseef et al., 2023). The instrument was selected over other methods as it separates commitment, execution, and outcome, so weakness in deployment or results is recorded explicitly rather than hidden inside a single holistic judgment. It also preserves comparability with the prior cybersecurity and software security readiness models. The three dimensions are as follows.

- **Approach:** evaluates organizational commitment, management support, and strategic planning for the practice. This dimension addresses whether the organization has consciously committed to the practice.
- **Deployment:** assesses the breadth, consistency, and comprehensiveness of practice execution across the organization. This dimension addresses the extent to which the practice is actually implemented.
- **Results:** evaluates the demonstrated effectiveness and measurable outcomes of the implemented practice. This dimension addresses whether the practice produces intended security improvements.

Each dimension is scored on a standardized scale (0, 2, 4, 6, 8, and 10), where 0 indicates no evidence of implementation and 10 indicates fully realized practice with continuous improvement, as shown in the scoring scale in the Supplementary material. The Motorola score for each practice is calculated in Equation 1 as the unweighted mean of the three dimensions:


$$\begin{array}{l} \text{Motorola Score}=\frac{\text{Approach}+\text{Deployment}+\text{Results}}{3} \end{array}$$ (1)

Each dimension is scored on the 0–10 scale, so the practice score is a normalized value from 0 to 10 representing implementation completeness. The dimensions are equally weighted, consistent with the original instrument (Daskalantonakis, 1994). Equal weighting is retained for two reasons. The three dimensions are not competing priorities to be traded off but successive conditions of an effective practice (commitment, execution, and outcome). Equal weights also preserve comparability with the readiness models that use the same instrument (Mufti et al., 2018; Humayun et al., 2023; Alnaseef et al., 2023).

#### 4.5.2 Advancement gates and level progression

A gate criterion governs progression between readiness levels. To advance to the next level, an organization must achieve an average Motorola score of at least 7.0 across all practices at its current level. This threshold follows prior readiness models employing the Motorola methodology (Mufti et al., 2018; Humayun et al., 2023; Alnaseef et al., 2023). The scoring anchors provide an operational interpretation of the threshold: 6 denotes “Marginally qualified” and 8 denotes “Qualified,” placing 7.0 at their midpoint. Like any cut-off imposed on a continuous scale, however, the threshold is a decision convention inherited from prior readiness models rather than an empirically established discontinuity. The gate also enforces sequential progression; an organization cannot claim a higher readiness level without passing all preceding levels. The C3RI implements this gate through an automated assessment tool. Assessors enter Approach, Deployment, and Results ratings for each practice, and the tool calculates the corresponding Motorola scores, overall level means, knowledge-area means, and advancement decisions. Because high-scoring practices can offset lower-scoring practices within a level mean, the tool preserves diagnostic visibility by reporting every practice score and flagging practices scoring at or below the 6.0 (“Marginally qualified”) anchor. These results allow weaknesses to be prioritized for remediation regardless of the advancement decision. The assessment tool is available in the companion repository.

Because level means close to 7.0 may be sensitive to small changes in individual ratings, a downgrade margin is reported as a supplementary stability indicator. The downgrade margin is the number of minimum one-step scoring downgrades required to reduce a level mean below 7.0, where one step represents a two-point reduction in one Motorola dimension for one practice. This indicator describes the sensitivity of the observed gate outcome to individual scoring changes; it does not alter the ≥7.0 advancement criterion or estimate assessor measurement error.

### 4.6 Knowledge area mapping reliability

To assess the reliability of the practice-to-CKA mapping produced during model development, we conducted an inter-coder agreement study using four independent large language model (LLM) agents as automated coders. This procedure complements the manual thematic analysis by quantifying how consistently the eight-CKA scheme can be applied to the practices, providing a supplementary assessment of the classification consistency underlying RQ2. We present this analysis as a supplementary, automated consistency check on the mapping. All agents were blind to the authors' original assignment. To keep disagreements meaningful, the agents were given opposed tie-breaking dispositions. Agent A applied the area names strictly and literally, resolving any multi-area practice toward the single area that most narrowly matches the control it describes, whereas Agent B reasoned about each practice's primary security objective and resolved multi-area practices toward the area that best reflects its overall purpose. Agents A and B ran on Claude Opus 4.8 (Anthropic). Because two coders that share one base model also share its priors about security taxonomies, the same two prompts were run, unchanged, on a second model family. Agents C (strict-literal) and D (reasoning-first) ran on GPT-5.6 Sol (OpenAI). Both runs used the provider-default decoding parameters.

The agents received the same 117-practice input, one entry per line, in the form CC-BP-N followed by the full practice text. Each agent was given only the practice text and the eight knowledge-area names. No definitions, keywords, or examples were supplied. We measured agreement with Cohen's κ (Cohen, 1960) and summarized multi-coder agreement using Fleiss' κ (Fleiss, 1971). The resulting values were interpreted against the benchmarks of Landis and Koch (1977).

Table 4 reports the results. The two Claude agents agreed with each other on 107 of 117 practices (κ = 0.90), and each agreed with the authors' mapping at κ = 0.83 (Agent A) and κ = 0.81 (Agent B), which is almost perfect agreement on the scale of Landis and Koch. The two GPT agents agreed with each other on 113 of 117 practices (κ = 0.96) and with the authors' mapping at κ = 0.76 (Agent C) and κ = 0.80 (Agent D), which is substantial agreement. The four cross-family pairs agreed at κ = 0.83–0.87. Fleiss' κ across the four agents is 0.87, and across all five coders including the authors 0.84. All five coders assigned the same CKA to 86 of 117 practices (73.5%), and the four agents were unanimous on 95 (81.2%).

**Table 4:** Inter-coder agreement for the practice-to-CKA mapping.

Coder pair	Observed agreement	Cohen's κ	95% CI	Interpretation
Agent A vs. authors	85.5%	0.83	[0.75, 0.90]	Almost perfect
Agent B vs. authors	83.8%	0.81	[0.73, 0.89]	Almost perfect
Agent C vs. authors	79.5%	0.76	[0.67, 0.84]	Substantial
Agent D vs. authors	82.9%	0.80	[0.71, 0.88]	Substantial
Agent A vs. Agent B (within family, Claude)	91.5%	0.90	[0.84, 0.95]	Almost perfect
Agent C vs. Agent D (within family, GPT)	96.6%	0.96	[0.92, 0.99]	Almost perfect
Agent A vs. Agent C	87.2%	0.85	[0.77, 0.92]	Almost perfect
Agent A vs. Agent D	88.9%	0.87	[0.79, 0.93]	Almost perfect
Agent B vs. Agent C	85.5%	0.83	[0.75, 0.90]	Almost perfect
Agent B vs. Agent D	87.2%	0.85	[0.77, 0.92]	Almost perfect
Fleiss' κ, four agents	–	0.87	[0.82, 0.92]	Almost perfect
Fleiss' κ, all five coders	–	0.84	[0.79, 0.89]	Almost perfect

The coefficients carry different evidential weight, and the pattern across them is informative. Within-family agreement is highest (κ = 0.90 for Claude, 0.96 for GPT), cross-family agreement is lower (0.83–0.87), and agreement with the human mapping is stable across families (0.76–0.83). The agent-to-author values are the primary evidence, because they compare an automated coder with the human thematic analysis that produced the mapping, and the fact that a second, unrelated model family reproduces them shows that they are not an artifact of one model's priors. The results indicate that the eight-area scheme can be re-applied from the area names alone, by coders with no access to the authors' reasoning, with substantial to almost-perfect agreement. What it does not establish is agreement among independent human experts, whose priors are not those of any language model.

Where coders diverged from the authors' mapping, the divergence was recorded and the practice retained under its primary CKA by author consensus. The divergences are systematic rather than random. All four coders placed machine-learning anomaly detection over urban datasets (CC-BP-69) in Monitoring and Response and the authentication practices protecting machine-learning pipelines (CC-BP-32, CC-BP-36) in Access Control and Authentication, in each case coding the practice by its mechanism, whereas the authors coded it by the asset whose security objective it serves. Bootstrap 95% confidence intervals for all agreement coefficients are reported in Table 4. The agent prompts, run metadata, confusion matrix, and the per-practice assignments of all five coders, with secondary CKA tags, are released in the companion repository.

## 5 Validation

We evaluated the C3RI in two complementary ways, namely an expert review by ten cybersecurity specialists, which provides the content-validation evidence, and an initial application case study in a real-world cognitive city context. A single case study or expert evaluation alone is unlikely to surface all of a model's deficiencies, so both were combined to obtain clearer evidence for RQ3. Figure 3 shows the validation process.

**Figure 3:**
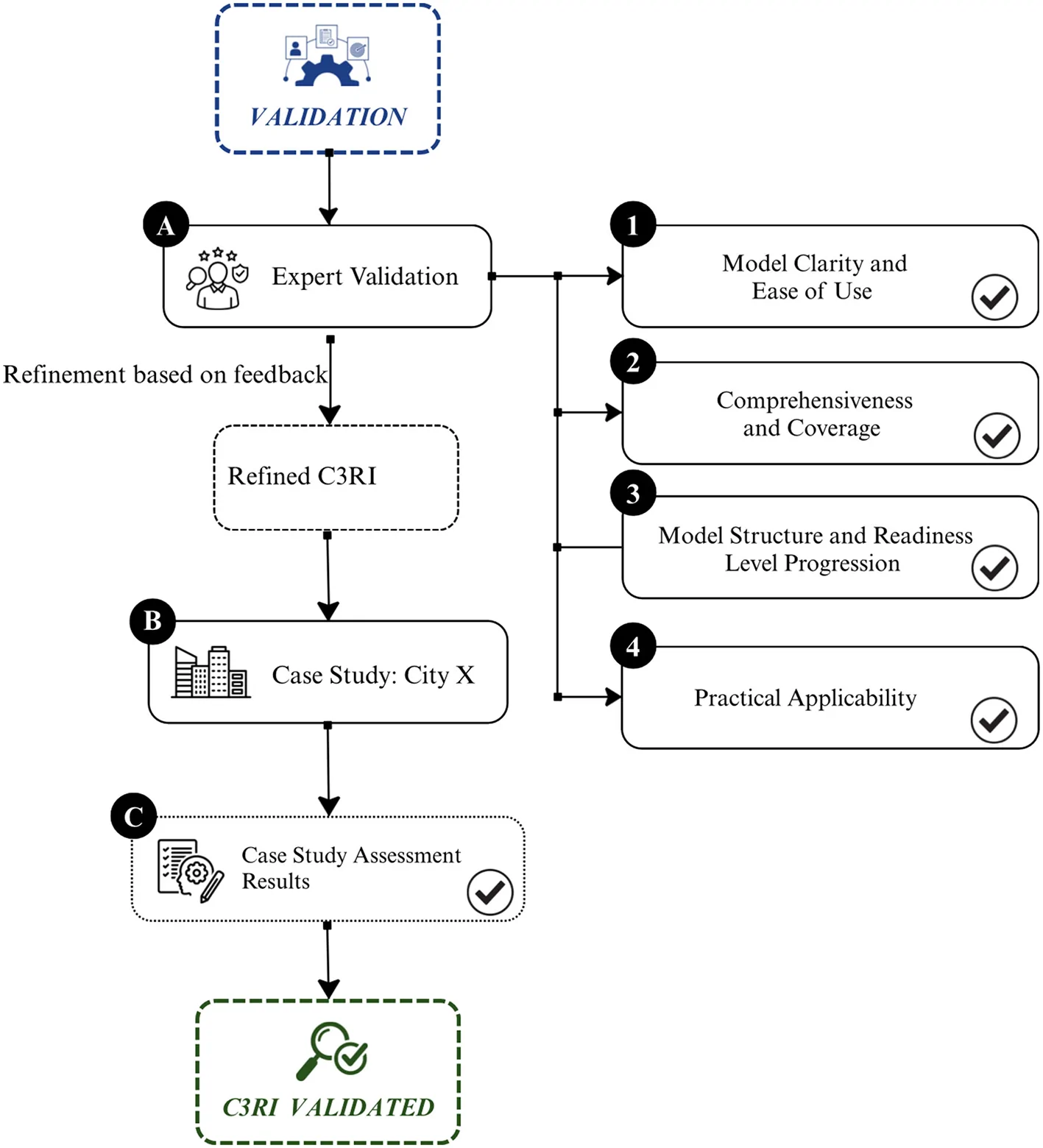
Validation process.

### 5.1 Expert review

An expert panel was recruited through purposive sampling using two eligibility criteria: relevant professional experience and a graduate-level qualification. Potential participants were identified through academic networks, professional cybersecurity conferences, and smart-city forums. Because cognitive-city cybersecurity is an emerging and specialized field, eligibility included substantive experience in closely related domains, such as smart-city cybersecurity, IoT security, and cyber-physical systems security. These domains share important technological, infrastructural, and organizational characteristics with cognitive-city environments. All participants held a graduate-level qualification in cybersecurity, computer science, or a related technical field and had at least four years of relevant professional experience. The final panel comprised ten experts (*n* = 10) with five academic researchers, two industry practitioners, and three government cybersecurity professionals. None was an author of this paper, belonged to the authors' research groups, or participated in developing the model. The panel therefore provided representation from research, industry, and government. Collectively, the industry and government participants covered operational functions including threat intelligence, incident response, and security operations center (SOC) monitoring. Table 5 summarizes the panel composition.

**Table 5:** Expert panel composition (*n* = 10).

ID	Role	Sector	Experience (years)	Highest qualification
E1	Academic researcher	Academia	10	PhD, Software Engineering
E2	Academic researcher	Academia	14	PhD, Computer Science
E3	Academic researcher	Academia	8	PhD, Computer Science
E4	Academic researcher	Academia	7	PhD, Cybersecurity
E5	Academic researcher	Academia	11	MSc, Computer Science
E6	Threat intelligence analyst	Government	5	MSc, Information Systems
E7	Incident responder	Government	6	MSc, Computer Science
E8	Incident responder	Government	5	MSc, Cybersecurity
E9	SOC analyst	Industry	4	MSc, Cybersecurity
E10	SOC analyst	Industry	4	MSc, Computer Science

The target panel size was established before recruitment based on guidance for expert-panel studies, whose purpose is to evaluate the content of a model rather than estimate a population parameter. Lynn (1986) recommends a minimum of three experts and up to ten to obtain stable judgments. Panels used to validate comparable readiness models (Mufti et al., 2018; Humayun et al., 2023; Alnaseef et al., 2023; AlGhamdi et al., 2024) were of similar or smaller size. The experts received the complete model documentation and evaluation survey. The survey contained nine items covering four dimensions: model clarity and ease of use; coverage of cognitive-city security requirements; organization and progression of the readiness levels; and practical applicability. Responses were recorded on a five-point Likert scale ranging from Strongly Disagree to Strongly Agree. For each item, endorsement was calculated as the proportion of experts selecting Agree or Strongly Agree. Open-ended responses were reviewed and grouped by topic, and every actionable suggestion was either implemented or documented with its resolution in the audit trail in the Supplementary material. Table 6 presents the full survey instrument and the response distributions.

**Table 6:** Expert evaluation results (*n* = 10).

	No. of participants = 10						
Survey question	SA	A	N	D	SD	Agreement (%)	Mdn
1. Every component in the model is self-explanatory and requires no additional clarification for adequate utilization.	3	7	0	0	0	100%	4
2. Are there any practices that need to be simplified or made clearer (If you agree, please specify).	0	2	0	8	0	80%^r^	2
3. The best practices are well-distributed among the eight knowledge areas and adequately cover cognitive city security requirements.	2	8	0	0	0	100%	4
4. The C3RI framework is comprehensive and complete for assessing cybersecurity readiness in cognitive cities.	1	8	1	0	0	90%	4
5. Do any of the practices seem unnecessary or redundant (If you agree, please specify).	0	1	0	6	3	90%^r^	2
6. The five readiness levels provide valuable structure for assessing cybersecurity capabilities in cognitive cities.	4	6	0	0	0	100%	4
7. Can the C3RI be expanded to include new practices as cognitive city technologies evolve.	3	7	0	0	0	100%	4
8. The C3RI framework can be effectively utilized to identify security weaknesses and gaps in cognitive cities.	4	6	0	0	0	100%	4
9. The C3RI framework is practical and can be feasibly applied by countries and organizations planning cognitive city infrastructure.	2	8	0	0	0	100%	4

SA, strongly agree; A, Agree; N, Neutral; D, Disagree; SD, strongly disagree.^r^reverse-scored item. Mdn, median.

#### 5.1.1 Model clarity and ease of use

All ten experts agreed that the model components are self-explanatory and require no additional clarification, with seven selecting Agree and three selecting Strongly Agree (mean = 4.30). When asked whether any practices required simplification, eight experts disagreed, and two agreed that some simplification might be helpful. Based on this feedback, four practices were reworded, as detailed in Section 5.3, to improve clarity. One expert also noted that formal training is not a prerequisite for applying the C3RI, suggesting that the model supports independent adoption.

#### 5.1.2 Comprehensiveness and coverage

All 10 experts agreed that the practices are well-distributed across the eight CKAs and adequately cover cognitive city security requirements, with eight selecting Agree and two selecting Strongly Agree (mean = 4.20). Regarding overall completeness, nine experts agreed that the C3RI is comprehensive for assessing cybersecurity readiness in cognitive cities, with one expert remaining neutral (mean = 4.00). The respondent with the neutral response suggested adding a practice on adversarial testing, which was subsequently incorporated as practice CC-BP-117. When asked directly whether any practices appeared unnecessary or redundant, six experts disagreed, and three strongly disagreed. However, one expert agreed that some practices might be redundant and specified them. In response to the expert feedback, we consolidated practices that addressed similar overlapping aspects of zero-trust architecture at the structured level, streamlining the model while preserving full coverage.

#### 5.1.3 Model structure and readiness level progression

The five-level readiness structure received a high level of agreement across all evaluated dimensions. All ten experts agreed that the readiness levels provide valuable structure for assessing cybersecurity capabilities, with four selecting Strongly Agree and six selecting Agree (mean = 4.40). This result suggests that the readiness level design conveys meaningful and distinguishable stages of readiness advancement, and that cities can recognize their current readiness within the model and understand the requirements for progression. This matters because the value of such models depends largely on whether they offer a clear structure, clear evaluation criteria, and practical methods for identifying strengths and areas for improvement (Alshareef et al., 2025). Regarding expandability, all ten experts agreed that the C3RI architecture can accommodate new practices as cognitive city technologies evolve, with seven selecting Agree and three selecting Strongly Agree (mean = 4.30). This finding is important for long-term model relevance as it indicates that the CKA structure and level definitions are sufficiently flexible to absorb emerging requirements without requiring fundamental redesign.

#### 5.1.4 Practical applicability

Practical applicability produced positive responses across both items. All ten experts agreed that C3RI can effectively identify security weaknesses and gaps in cognitive city designs, with four selecting Strongly Agree and six selecting Agree (mean = 4.40). All ten experts also agreed that the model is practical and can be feasibly applied by countries and organizations planning cognitive city infrastructure, with two selecting Strongly Agree and eight selecting Agree (mean = 4.20). One expert stated that deep familiarity with the underlying assessment methodology is not required to apply the model effectively. Two points emerged from the open-ended feedback. First, several experts stressed the importance of building periodic review cycles into the model lifecycle so that practices keep pace with technological change in cognitive city deployments. Second, one expert noted that an automated tool can reduce the time and expertise required to conduct a full assessment.

#### 5.1.5 Expert agreement

Table 6 reports the proportion of model-favorable responses for each item. For the seven positively worded items (1, 3, 4, 6, 7, 8, and 9) this is the proportion selecting Agree or Strongly Agree; for the two items asking whether practices need simplification (item 2) or are redundant (item 5), it is the proportion selecting Disagree or Strongly Disagree. Six items (1, 3, 6, 7, 8, and 9) reach 100%, items 4 and 5 reach 90%, and item 2 reaches 80%. To explore whether professional background influenced the expert evaluation, responses were examined descriptively across academic (*n* = 5), government (*n* = 3), and industry (*n* = 2) participants, focusing on comprehensiveness (Q4), readiness-level structure (Q6), gap identification (Q8), and practical applicability (Q9). All participants across the three professional groups provided positive responses (Agree or Strongly Agree) to Q6, Q8, and Q9. For Q4, all government and industry participants and four academic participants provided positive responses, and only one academic participant selected Neutral. Thus, no systematic divergence in positive versus non-positive responses was apparent across professional groups. Given the panel size (*n* = 10) and the unequal subgroup sizes, this comparison is descriptive, and no inferential claim is made regarding professional background effects.

### 5.2 Case study: City X

The case study was conducted in City X, a large-scale national cognitive city development initiative encompassing smart governance systems, autonomous decision-making platforms, distributed IoT networks spanning multiple urban service domains, integrated data analytics infrastructure, and AI-driven citizen service interfaces. The city had progressed beyond planning, with established security practices in place, but was not yet fully operational, which is precisely the phase at which the C3RI is designed to provide actionable guidance. The finalized 117-practice version was used in the City X assessment. Due to confidentiality and non-disclosure agreements, the city's identity is anonymized throughout this paper as City X.

#### 5.2.1 Case study assessment results

The assessment of City X was conducted across all 117 best practices using the Motorola-based assessment methodology. The assessment was led by a cybersecurity expert with more than 20 years of experience in ICT infrastructure and smart cities, working with four members of the city's cybersecurity team responsible for the relevant systems and controls. Scoring was conducted in working sessions in which each practice was rated against the city's documentary evidence, technical evidence, and the operational knowledge of the responsible team members. The lead expert served as the single finalizing respondent for the Approach, Deployment, and Results scores. Because City X had progressed beyond planning but was not yet fully operational, the Results dimension was scored against the evidence available at this stage, including partially implemented controls and outcomes from design and pilot activities, rather than long-run operational metrics. Table 7 presents the level-by-level results.

**Table 7:** C3RI Assessment results for City X.

Readiness level	Practices	Motorola score	Gate status
Level 1: Foundational	31	7.27	PASS
Level 2: Structured	37	7.28	PASS
Level 3: Intelligent	22	7.52	PASS
Level 4: Autonomous	11	7.15	PASS
Level 5: Adaptive	16	7.04	PASS
Mean of level means	-	7.25	All means ≥ 7.0

City X's level means exceed the 7.0 advancement gate at all five levels. The highest score, 7.52 at Level 3 (Intelligent), indicates that City X has invested substantially in AI-assisted threat detection, predictive analytics, and intelligent security recommendations. The relatively consistent scores across levels indicate that City X has pursued a broadly balanced security implementation rather than concentrating strength in any single level. The level means nevertheless conceal important variation among individual practices and knowledge areas. Practice scores ranged from 5.33 to 9.33, and 49 of the 117 practices (41.9%) scored below 7.0. Knowledge-area means ranged from 6.91 for Training and Awareness to 7.67 for Access Control and Authentication, as shown in Table 8. These differences illustrate the instrument's ability to identify areas of relative strength and weakness that are not apparent from the overall level means. Conducting the assessment during the design and construction phases enabled City X's leadership to identify cybersecurity gaps before full deployment. The case therefore illustrates the potential value of applying the C3RI during infrastructure development rather than deferring assessment until systems become operational. Following the assessment, the lead expert proposed two improvements to the model's usability. First, the model should use a simplified visual presentation with color-coded status indicators to help practitioners identify practices requiring attention. Second, a web-based assessment tool should automate scoring, retain historical results, and issue reminders when reassessments are due.

**Table 8:** City X assessment by Cybersecurity knowledge area (sorted by mean Motorola score).

Knowledge area	Practices	Mean score	Lowest practice	Practices < 7.0
Training & awareness	11	6.91	5.33	5
Governance & compliance	13	7.03	5.33	8
Monitoring & response	27	7.19	6.00	13
Data protection & privacy	15	7.29	5.33	8
Network & infrastructure	17	7.41	6.67	6
Vendor & supply chain	8	7.42	6.67	2
AI/ML security	18	7.44	5.33	5
Access control & authentication	8	7.67	6.67	2
All practices	117	7.28	5.33	49

#### 5.2.2 Robustness of the advancement gate

Because several level means lie close to the 7.0 threshold, two analyses probe the observed gate outcomes, summarized in Table 9. The first concerns the evidence basis of the Results dimension. A pre-operational assessment yields provisional Results ratings, so rather than defining a separate pre-operational scoring scheme, the C3RI reports the level means recomputed from Approach and Deployment alone as the pre-operational view of the gate outcome. For City X, these recomputed means were 7.23, 7.41, 7.55, 7.36, and 7.38 for Levels 1–5, so all five levels remain above the 7.0 threshold and no advancement decision depends on the provisional Results ratings. This test does not substitute for independently verified operational Results, which only reassessment of City X can provide when it becomes fully operational. The second concerns scoring uncertainty. The downgrade margin defined in Section 4.5.2 was 13, 16, 18, 3, and 2 for Levels 1–5, respectively. The advancement decisions for Levels 1–3 are therefore relatively insensitive to isolated scoring changes, whereas Levels 4 and 5 lie closer to the advancement boundary and should be interpreted with greater caution.

**Table 9:** Robustness of the City X gate outcome.

Level	*n*	Mean	SD	A	D	R	A+D only	Margin
L1	31	7.27	0.95	7.10	7.35	7.35	7.23	13
L2	37	7.28	0.92	7.46	7.35	7.03	7.41	16
L3	22	7.52	0.88	7.27	7.82	7.45	7.55	18
L4	11	7.15	0.85	7.45	7.27	6.73	7.36	3
L5	16	7.04	0.64	7.62	7.12	6.38	7.38	2

A, approach; D, deployment; R, results.

Margin: number of single two-point downgrades that would take the level mean below 7.0.

### 5.3 C3RI modifications

Following the experts' feedback, we undertook a structured review to refine the model. The refinements were completed before the City X assessment, which applied the final 117-practice set and produced usability recommendations rather than changes to practice content. Four practices (CC-BP-21, CC-BP-42, CC-BP-51, and CC-BP-87) received targeted wording clarifications to make assessment interpretation more consistent. In CC-BP-21, the ROSI abbreviation was expanded to “Return on Security Investment;” CC-BP-42 was clarified around “context-aware identity and access management;” CC-BP-51 gained more prescriptive implementation examples for “dedicated security controls to fog computing nodes;” and CC-BP-87 was strengthened to state explicitly that critical security events must be delivered immediately. Expert feedback also flagged potential redundancy among the zero-trust practices at the Structured level: a review of CC-BP-40 and related practices revealed overlapping coverage of identity verification and access restriction, which we consolidated while preserving full coverage. This consolidation reduced the practice set from 121 to 116. Finally, one expert suggested adding a practice on formal adversarial testing at Level 5 (Adaptive), which we added as CC-BP-117 in the Monitoring and Response knowledge area. With the addition of CC-BP-117, the final model comprises 117 practices. Expert respondents recommended operationalizing the C3RI assessment through an automated tool, which we created and uploaded in the accompanying repository. This tool reduces data-entry error by validating score ranges, calculating Motorola averages, and evaluating advancement-gate status for each readiness level. We also present the C3RI model through clear visual representations, including practice-distribution tables by readiness level and knowledge area. In response to City X's recommendation, we also plan to develop a web-based assessment platform with an integrated database, so that organizations can conduct assessments through guided, interactive forms with real-time validation. The platform will store and track historical assessment results to measure readiness progress and will generate remediation roadmaps with prioritized actions and investment recommendations. This will further reduce the assessment burden, improve data consistency, and give decision-makers clear visibility into how cybersecurity readiness progresses over time.

## 6 Discussion

Maturity models and readiness models play distinct roles in organizational assessment. A maturity model characterizes an organization's current state and evaluates its processes and practices to pinpoint areas that need improvement (Ünlü et al., 2024; Schumacher et al., 2016). A readiness model, in contrast, assesses whether an organization is prepared to adopt or undertake a new process or transformation before that effort begins (Schumacher et al., 2016). Cognitive cities are not merely more advanced smart cities; they raise security challenges that are qualitatively different from those existing frameworks were designed to handle. Vitunskaite et al. (2019), for example, observed that conventional cybersecurity standards and frameworks do not directly address the security requirements of smart cities, a gap that threatens the functionality of urban infrastructure. The C3RI closes the gap with a structured, evidence-based readiness model built for the cognitive city context. It assumes a baseline of conventional organizational cybersecurity and assesses readiness for cognitive-city-specific capabilities; it is not a replacement for a general controls catalog. The distinction between readiness and maturity presented in Section 4 is primarily a design distinction. The case study illustrates the intended readiness interpretation because the C3RI could be applied before City X became fully operational, using documentary, technical, design, and pilot evidence. This demonstrates the feasibility of pre-operational assessment but does not establish mature operational performance at all five levels. Empirical examination of the distinction through comparative application of the C3RI and an established maturity instrument remains an area for future work. The C3RI was developed through a systematic methodology that pairs a focused literature review with empirical evaluation through an independent expert review and an initial real-world application case study. This mirrors how comparable domain-specific readiness models have been built in adjacent fields. Humayun et al. (2023) developed a readiness model for secure software coding from literature-grounded practice identification, expert validation, and organizational case studies, and argued that systematic development yields models more credible than those built on informal expert opinion alone. Mufti et al. (2018) took the same approach for a security requirements engineering readiness model; they validated it across several organizations and concluded that systematic development and expert review are essential to a model that organizations can trust and apply. Yigit Ozkan et al. (2021) validated the CYSFAM framework with a case study and expert interviews, and reported that both methods were needed to surface deficiencies that neither could reveal alone. The C3RI carries this approach into the cognitive city domain and is, to our knowledge, the first evidence-based, security-specific readiness model tailored to the threats of autonomous, AI-driven urban systems. As in those prior models, it relies on the Motorola assessment method and a level-based advancement gate to give a transparent, reproducible measure of cybersecurity readiness.

The City X case study produced relatively similar mean scores across the five readiness levels, ranging from 7.04 to 7.52. The stability analysis suggests that the advancement decisions for Levels 1–3 are less sensitive to isolated one-step scoring changes, whereas Levels 4 and 5 are closer to the advancement threshold and should therefore be interpreted more cautiously. Because operational cognitive city infrastructure remains scarce worldwide, the study was limited to a single case. A comparable model faced the same constraint; Yigit Ozkan et al. (2021) validated CYSFAM with a single in-depth case study supplemented by expert interviews and treated depth rather than breadth as the basis for rigor.

The expert panel (*n* = 10) reinforced the case-study findings. Positive responses across all nine survey items, with the strongest consensus on the readiness-level structure and practical applicability, suggest that domain experts regard the C3RI as technically sound and deployable, although with *n* = 10 this is descriptive, exploratory evidence of expert opinion rather than an empirical demonstration. Together, the initial application case study and the expert review give initial confidence in the C3RI's applicability for assessing cybersecurity readiness in cognitive cities. The model also rests on a knowledge-area scheme whose consistency was probed by a supplementary automated check, as reported in Section 4.6. Four LLM agents from two model families, working from the area names alone, reproduced the authors' practice-to-CKA mapping with κ of 0.76–0.83, indicating that the eight knowledge areas can be applied consistently from the CKA names alone and that this does not depend on the priors of one model. This is a consistency check rather than external validation, and agreement among independent human experts remains to be established.

## 7 Limitations

This study has several limitations, each with the mitigation applied and the validation step that addresses it. First, the empirical evaluation rested on a single case study, because operational cognitive city implementations remain few worldwide, so the results should not be assumed to generalize to cities at other stages of deployment. We mitigated this with the expert panel of ten cybersecurity specialists from academia, industry, and government, who assessed the model independently. A single case also limits assessment of discriminatory validity between cities. Although the City X case demonstrates that C3RI produces differentiated practice, knowledge area, and level-specific scores within one city, cross-city discriminatory validity cannot be established without comparative assessments of cities with genuinely different readiness profiles. Furthermore, City X was assessed through a team-supported, evidence-based process with one finalizing respondent, because confidentiality constraints did not permit independent parallel ratings. A single-respondent design carries risks of common-method bias, subjective scoring, and institutional-desirability bias that the documentary and technical evidence review mitigates but cannot eliminate, and rater error could not be estimated directly. The Results scores also rest partly on design and pilot evidence. Excluding the Results dimension did not change any advancement decision, but this sensitivity analysis cannot establish what the Results ratings will be after full deployment. Longitudinal reassessment using operational outcome evidence is therefore required.

A second limitation concerns the expert review, in both its size and its form. While the panel size is consistent with guidance on expert-panel size, it is too small for inferential comparison of ratings across professional backgrounds, and a larger, more diverse panel would enable such comparisons and broaden the evidence of the model's applicability across practice contexts. The review also elicited judgments about the model as a whole, namely its clarity, coverage, structure, and practical applicability, rather than relevance ratings for each of the 117 practices. A formal content-validity study, in which an independent panel rates every practice for relevance, remains future work. A third limitation concerns the reliability check of the knowledge-area mapping, in which LLM agents were used as automated coders, not independent human raters. The inclusion of two model families reduces the likelihood that the observed agreement was specific to one model architecture or set of weights, but it does not eliminate the possibility of shared priors arising from similar training corpora. The agents reproduced the authors' mapping with substantial to almost-perfect agreement, but confirming this with a panel of independent human coders remains useful future work. Finally, the rapid pace of technological change in cognitive cities, particularly in AI governance and autonomous systems security, calls for a structured review cycle to ensure the model's continued relevance as deployments mature and the threat environment evolves. The C3RI is intended for cognitive cities that differ in regulatory environment, infrastructure architecture, level of AI adoption, and threat profile. Establishing external validity across that range requires a validation strategy. We propose the following strategy, which the open assessment tool and data are designed to support:

- Multi-site replication using maximum-variation sampling to select cities that differ deliberately in regulatory environment, infrastructure architecture, degree of AI adoption, and threat profile.
- Known-groups comparisons testing whether cities at clearly different deployment stages (planning, construction, partial operation, and full operation) produce the hypothesized differences in C3RI outcomes.
- Multi-rater assessment within each city using at least two independent assessors to estimate inter-rater reliability for the Motorola dimensions and examine the stability of advancement decisions. Score distributions from multiple cases could also inform calibration of the 7.0 advancement threshold and test advancement classifications under alternative thresholds and weighting schemes.
- Longitudinal reassessment of the same cities at intervals of 12 to 18 months to determine whether pre-operational readiness assessments are supported by subsequent operational evidence and whether C3RI scores respond appropriately to documented changes in capability.
- Regional content re-validation in which expert panels from each regulatory region evaluate the practice library for locally required additions or inapplicable practices while retaining a common core level and knowledge-area structure to preserve cross-regional comparability.

Together, these steps would evaluate the model's generalizability, inter-rater reliability, predictive validity, and responsiveness to change. The structured review cycle would provide a mechanism for incorporating the resulting evidence into future versions of the C3RI.

## 8 Conclusions

Cognitive cities represent an evolution in urban infrastructure through their integration of advanced AI, autonomous systems, and complex IoT ecosystems. This combination introduces security challenges that require assessment methods tailored to the cognitive-city context. This paper addresses that need by developing the Cognitive City Cybersecurity Readiness Index (C3RI), to our knowledge, the first security-specific readiness model designed for cognitive cities. The model was developed through a structured methodology combining a focused review of peer-reviewed literature and international standards with iterative refinement. The resulting framework comprises 117 literature- and standards-grounded security practices organized into eight Cybersecurity Knowledge Areas and five progressive readiness levels.

The Motorola-based assessment methodology evaluates each practice across the Approach, Deployment, and Results dimensions and applies a level-mean advancement threshold of 7.0. This structure provides cognitive-city initiatives with a transparent and reproducible means of identifying weakly implemented practices and prioritizing cybersecurity investment. An initial real-world application case study provided preliminary evidence of the model's practical applicability. A panel of ten domain experts provided complementary, favorable assessments of its clarity, coverage, readiness-level structure, and practical applicability. The knowledge-area scheme underlying the model was also subjected to a supplementary automated consistency check. Four LLM-based classifiers from two model families agreed with the authors' practice-to-CKA mapping at κ = 0.76–0.83. This result provides preliminary evidence that the knowledge-area labels capture distinctions that the tested models could recover, but it does not replace validation by independent human coders.

The C3RI offers cities, organizations, and policymakers structured guidance for developing cybersecurity capabilities before and during cognitive-city deployment. The current case study, expert review, and automated consistency check provide preliminary rather than generalizable validation. Future research should implement the validation strategy outlined in Section 7, including longitudinal and multi-rater assessments of heterogeneous cognitive-city implementations across geographic and regulatory contexts. Structured review cycles will also be required to keep the model aligned with evolving technologies, operational evidence, and emerging threats.

## Data Availability

The datasets presented in this study can be found in online repositories. The names of the repository/repositories and accession number(s) can be found in the article/Supplementary material.
