# Supplementary material for: The Cognitive City Cybersecurity Readiness Index (C3RI): an evidence-based model for AI-driven urban infrastructure
Source: Front Big Data. 2026 Oct 28;9:1949829. doi: 10.3389/fdata.2026.1949829 (PMC13752211; doi:10.3389/fdata.2026.1949829)
Supplement: Supplementary file 1 [file Supplementary_file_1.pdf]

Supplementary Material

This Supplementary Material accompanies the article “The Cognitive City Cybersecurity Readiness Index (C3RI): an evidence-based model for AI-driven urban infrastructure” (Front. Big Data 9:1949829). It contains three supplementary items, namely the complete C3RI best practice library organised by readiness level (Appendix A), the Motorola assessment instrument used to score each practice (Appendix B), and the audit trail of every change made to the practice set during model refinement (Appendix C).

Appendix A: C3RI Best Practices by Readiness Level

Table A1: Level 1 (Foundational) practices.

Practice ID	Best practice	Knowledge area
CC-BP-1	Ensure machine learning models and AI algorithms are secure by design with explainability for validation	AI/ML Security
CC-BP-2	Conduct thorough security assessments of externally sourced AI before urban integration	AI/ML Security
CC-BP-3	Prepare specific procedures for handling security incidents involving AI-powered city services	AI/ML Security
CC-BP-4	Address bias and integrity defects in training data that degrade model reliability and produce discriminatory outcomes in city services	AI/ML Security
CC-BP-5	Implement security-by-design for all sensors and devices contributing to AI training datasets	AI/ML Security
CC-BP-6	Maintain documented explainability mechanisms for all AI decisions affecting security operations	AI/ML Security
CC-BP-7	Assess security of all cognitive sensors feeding data into predictive and learning systems	Data Protection & Privacy
CC-BP-8	Define data retention policies that balance AI training needs with privacy requirements	Data Protection & Privacy
CC-BP-9	Conduct privacy impact assessments for cognitive systems processing personally identifiable information	Data Protection & Privacy
CC-BP-10	Establish purpose specification protocols ensuring collected data serves only explicitly defined cognitive functions with citizen consent	Data Protection & Privacy
CC-BP-11	Establish data minimization protocols to use only necessary data for cognitive decisions	Data Protection & Privacy
CC-BP-12	Conduct a comprehensive inventory of all cognitive city assets (including sensors, edge devices, AI platforms, and data lakes)	Governance & Compliance
CC-BP-13	Develop procedures for switching from autonomous to manual operation during cyber incidents	Governance & Compliance
CC-BP-14	Document clear accountability chains for all AI-driven decisions, data flows, and incident response actions	Governance & Compliance
CC-BP-15	Establish clear data ownership and usage policies for information collected by cognitive infrastructure	Governance & Compliance
CC-BP-16	Verify that local AI governance policies are aligned with applicable national cybersecurity and AI ethics standards	Governance & Compliance
CC-BP-17	Conduct comprehensive cybersecurity risk analysis and threat modeling early in the design and planning stages	Governance & Compliance
CC-BP-18	Establish security standards for interoperability across cognitive city platforms	Governance & Compliance
CC-BP-19	Integrate security-by-design principles into project planning and bids from inception to preempt retrofit costs	Governance & Compliance
CC-BP-20	Establish formal governance requiring cybersecurity expert participation from design through full deployment of all cognitive city systems	Governance & Compliance
CC-BP-21	Develop a cybersecurity investment roadmap prioritizing initiatives based on Return on Security Investment (ROSI) or equivalent methodology	Governance & Compliance
CC-BP-22	Establish and communicate a formal reporting channel for city staff and residents to report suspected security anomalies in cognitive city services	Training & Awareness
CC-BP-23	Establish cognitive cybersecurity education across schools and training centers for future workforce	Training & Awareness
CC-BP-24	Establish periodic public awareness campaigns to educate citizens on cognitive city security and privacy	Training & Awareness
CC-BP-25	Develop a cybersecurity workforce plan with defined roles, skills gap analysis, and recruitment strategies	Training & Awareness
CC-BP-26	Develop and maintain plans for timely replacement of cognitive city components approaching or reaching end-of-life	Vendor & Supply Chain
CC-BP-27	Specify minimum security requirements clearly to all cognitive city vendors	Vendor & Supply Chain
CC-BP-28	Outline specific actions to take if vendors breach security requirements	Vendor & Supply Chain
CC-BP-29	Require vendors to follow a secure software development lifecycle process	Vendor & Supply Chain
CC-BP-30	Embed pre-defined security standards into all contractual agreements	Vendor & Supply Chain
CC-BP-31	Include explicit prohibitions on backdoors in vendor agreements, mandating their disablement or hardening post-deployment	Vendor & Supply Chain

Table A2: Level 2 (Structured) practices.

Practice ID	Best practice	Knowledge area
CC-BP-32	Deploy controls to prevent authentication bypass in machine learning pipelines	AI/ML Security
CC-BP-33	Create isolated zones for developing, training, validating, and operating AI systems in city infrastructure	AI/ML Security
CC-BP-34	Implement logical and physical isolation for security-critical AI components (e.g., decision engines, model weights, training algorithms, reward/loss functions for ML) from general operational systems	AI/ML Security
CC-BP-35	Create digital fingerprints for AI models deployed in city infrastructure to verify authenticity	AI/ML Security
CC-BP-36	Establish authentication protocols for devices to prevent compromised IoT sensors from poisoning AI data	AI/ML Security
CC-BP-37	Deploy input validation, model integrity monitoring, and adversarial robustness testing to protect AI models against poisoning and evasion attacks	AI/ML Security
CC-BP-38	Protect real-time AI inference pathways from interception or manipulation	AI/ML Security
CC-BP-39	Establish regular penetration testing for cognitive decision-making pathways	AI/ML Security
CC-BP-40	Deploy zero trust architecture across all cognitive system interconnections and data flows	Access Control & Authentication
CC-BP-41	Mandate multi-factor authentication (MFA) for all access to cognitive systems, AI control panels, and administrative interfaces	Access Control & Authentication
CC-BP-42	Implement role-based and context-aware identity and access management (IAM) to govern access to AI training data, models, and decision outputs	Access Control & Authentication
CC-BP-43	Create and test backups of AI models, training data, and control configurations	Data Protection & Privacy
CC-BP-44	Encrypt all data used to build, validate, and operate urban AI capabilities	Data Protection & Privacy
CC-BP-45	Validate the origin and authenticity of data before incorporating it into city AI systems	Data Protection & Privacy
CC-BP-46	Implement lightweight cryptographic algorithms (e.g., Ascon, PRESENT) optimized for resource-constrained IoT devices	Data Protection & Privacy
CC-BP-47	Classify urban data by sensitivity levels and enforce corresponding access restrictions	Data Protection & Privacy
CC-BP-48	Implement post-quantum cryptography (PQC) to protect against future quantum computing threats	Data Protection & Privacy
CC-BP-49	Deploy anonymization and de-identification at data collection before entering analytics pipelines	Data Protection & Privacy
CC-BP-50	Create regulatory sandboxes to test novel AI security approaches before citywide deployment	Governance & Compliance
CC-BP-51	Apply dedicated security controls to fog computing nodes (e.g., hardware attestation, encrypted local processing, and strict data minimization) before cloud transmission	Network & Infrastructure
CC-BP-52	Apply layered, defense-in-depth security approach with redundant measures across cognitive city infrastructure	Network & Infrastructure
CC-BP-53	Implement network micro-segmentation for cognitive city infrastructure with strict access controls for each AI module	Network & Infrastructure
CC-BP-54	Restrict lateral movement so compromised AI systems only affect their isolated segment	Network & Infrastructure
CC-BP-55	Apply model compression techniques (e.g., pruning, quantization) to deploy lightweight AI models on edge hardware	Network & Infrastructure
CC-BP-56	Adopt low-power, long-range wireless networks (LPWAN) that include robust security protocols	Network & Infrastructure
CC-BP-57	Deploy protocol translation gateways with cryptographic overlays to modernize obsolete protocols	Network & Infrastructure
CC-BP-58	Adopt secure lightweight protocols for IoT devices (e.g., CoAP with DTLS, MQTT over TLS)	Network & Infrastructure
CC-BP-59	Adopt compact encodings (e.g., CBOR) and header compression for IoT data transmission	Network & Infrastructure
CC-BP-60	Apply tiered security profiles (low/medium/high) tailored to device capabilities for consistent protection of heterogeneous IoT assets	Network & Infrastructure
CC-BP-61	Use adaptive duty cycling on low-power IoT devices	Network & Infrastructure
CC-BP-62	Deploy gateway offloading to shift compute-intensive security tasks	Network & Infrastructure
CC-BP-63	Use energy-harvesting technologies to preserve the availability of security-critical sensors and telemetry where physical access or battery replacement is impractical	Network & Infrastructure
CC-BP-64	Train personnel to monitor, validate, and override AI-driven decisions when necessary	Training & Awareness
CC-BP-65	Conduct collaborative cyber war-gaming exercises, large-scale simulations involving multiple stakeholders	Training & Awareness
CC-BP-66	Develop and deliver cognitive city-specific security training for personnel	Training & Awareness

Table A2 continued from previous page

Practice ID	Best practice	Knowledge area
CC-BP-67	Maintain and regularly update software bills of materials (SBOMs) for all cognitive system components	Vendor & Supply Chain
CC-BP-68	Create scenario-based resilience testing simulating coordinated attacks on interconnected systems	Governance & Compliance

Table A3: Level 3 (Intelligent) practices.

Practice ID	Best practice	Knowledge area
CC-BP-69	Deploy machine learning-based anomaly detection that continuously monitors urban datasets for manipulation, errors, corruption, and suspicious patterns before use	AI/ML Security
CC-BP-70	Use machine learning for predictive threat intelligence to identify attack patterns early	Monitoring & Response
CC-BP-71	Deploy machine-learning or analytics-based continuous validation that flags AI-output drift, poisoning, or anomalies	AI/ML Security
CC-BP-72	Enable threat hunting where intelligent agents continuously probe cognitive infrastructure for vulnerabilities	Monitoring & Response
CC-BP-73	Utilize digital twin simulations to model and predict cyberattack propagation, failure conditions, and system-wide impacts	Monitoring & Response
CC-BP-74	Implement identity and access management (IAM) with role-based access control that dynamically adjusts based on behavioral analytics	Access Control & Authentication
CC-BP-75	Dynamically adjust time-bound and context-aware privileges for access to cognitive infrastructure components	Access Control & Authentication
CC-BP-76	Require continuous authentication and authorization for AI-to-AI and human-to-AI interactions	Access Control & Authentication
CC-BP-77	Enforce continuous verification eliminating implicit trust for users, devices, apps, or AI agents	Access Control & Authentication
CC-BP-78	Add calibrated random noise to aggregated data outputs to obscure individual identities	Data Protection & Privacy
CC-BP-79	Implement blockchain-based audit trails with analytics for data access and AI decision provenance	Data Protection & Privacy
CC-BP-80	Automate compliance reporting against applicable cybersecurity standards covering all cognitive city infrastructure and AI systems	Governance & Compliance
CC-BP-81	Configure intelligent alerting that notifies security teams when AI systems deviate from expected operational patterns	Monitoring & Response
CC-BP-82	Establish AI-augmented integrated security operations centers (ISOC) monitoring IT, OT, AI, physical security, and insider threats	Monitoring & Response
CC-BP-83	Enable real-time threat intelligence sharing across all security domains to inform cognitive/AI-driven decision-making	Monitoring & Response
CC-BP-84	Integrate and aggregate security data from IT, OT, AI, networks, endpoints, and IoT for comprehensive real-time threat visibility	Monitoring & Response
CC-BP-85	Implement predictive risk scoring mechanisms for all connected IoT devices and infrastructure nodes	Monitoring & Response
CC-BP-86	Implement predictive maintenance for cybersecurity infrastructure to anticipate vulnerabilities	Monitoring & Response
CC-BP-87	Implement event-driven monitoring with batched telemetry uploads to optimize bandwidth utilization and energy consumption across sensor networks while ensuring critical security events are delivered immediately	Network & Infrastructure
CC-BP-88	Implement AI-powered virtual citizen labs offering gamified simulations of cognitive city cyberattacks that provide intelligent feedback to educate residents on recognizing and reporting AI-specific threats	Training & Awareness
CC-BP-89	Deploy ongoing monitoring for third-party technology partner risks	Vendor & Supply Chain
CC-BP-90	Deploy privacy-preserving AI techniques including federated learning, differential privacy, and homomorphic encryption	Data Protection & Privacy

Table A4: Level 4 (Autonomous) practices.

Practice ID	Best practice	Knowledge area
CC-BP-91	Build validation pipelines to continuously assess AI system integrity without manual intervention	AI/ML Security

Table A4 continued from previous page

Practice ID	Best practice	Knowledge area
CC-BP-92	Enable automatic rollback of compromised AI models to previous verified versions when inference anomalies exceed learned thresholds	AI/ML Security
CC-BP-93	Implement immediate role-based access revocation for anomalous users or AI agents interacting with IoT systems	Access Control & Authentication
CC-BP-94	Develop, test, and maintain autonomous incident response plans that automatically activate pre-defined countermeasures for defined attack scenarios	Monitoring & Response
CC-BP-95	Implement self-healing systems that automatically isolate compromised nodes and reroute services	Monitoring & Response
CC-BP-96	Create fallback modes to operate in safe-limited functionality if AI monitoring detects system compromise	Monitoring & Response
CC-BP-97	Deploy quarantine mechanisms that isolate compromised IoT devices when behavioral deviation exceeds defined thresholds	Monitoring & Response
CC-BP-98	Isolate critical cognitive infrastructure segments while enabling secure inter-system learning	Network & Infrastructure
CC-BP-99	Deploy automated patching processes with authenticity and integrity validation for all updates	Network & Infrastructure
CC-BP-100	Deploy micro-patching engines for IoT firmware that implements minimal-risk patches dynamically without requiring full firmware updates	Network & Infrastructure
CC-BP-101	Implement autonomous security awareness systems that automatically personalize training content based on user roles and emerging threats	Training & Awareness

Table A5: Level 5 (Adaptive) practices.

Practice ID	Best practice	Knowledge area
CC-BP-102	Deploy AI-driven forensics capabilities for post-incident learning and system improvement	Monitoring & Response
CC-BP-103	Deploy automated threat hunting mechanisms that learn from historical attack patterns	Monitoring & Response
CC-BP-104	Enable continuous threat model advancement where autonomous threat hunting agents generate new attack hypotheses	Monitoring & Response
CC-BP-105	Feed internal incident data, red team exercises, and attack patterns into automated threat model learning systems	Monitoring & Response
CC-BP-106	Implement learning-based threat model evolution where internal incident data automatically feeds updated threat scoring matrices	Monitoring & Response
CC-BP-107	Implement learning-based forensics where incident analysis results automatically generate new detection rules	Monitoring & Response
CC-BP-108	Deploy self-evolving threat models that automatically incorporate new attack vectors from global intelligence feeds	Monitoring & Response
CC-BP-109	Operationalize AI-enhanced forensics tools that continuously improve pattern recognition from cumulative incident data	Monitoring & Response
CC-BP-110	Use reinforcement learning to dynamically optimize IoT resource allocation during cyberattacks	Monitoring & Response
CC-BP-111	Integrate incident-driven learning outputs (forensics, threat hunting, threat models) into a unified adaptive defense orchestration process	Monitoring & Response
CC-BP-112	Implement continuous, adaptive security auditing and monitoring across all cognitive city infrastructure components	Monitoring & Response
CC-BP-113	Enable automatic security resource reallocation that dynamically provisions additional compute/bandwidth to high-risk cognitive nodes	Monitoring & Response
CC-BP-114	Implement adaptive anomaly detection thresholds that account for seasonal and event-based patterns in urban data	Monitoring & Response
CC-BP-115	Conduct collaborative cyber war-gaming exercises and automatically evolve threat models based on simulated attack outcomes	Training & Awareness
CC-BP-116	Continuously assess and automatically update cognitive city security role requirements and competency profiles based on the evolving threat landscape	Training & Awareness
CC-BP-117	Maintain autonomous red team/adversarial teams that continuously evolve attack simulations	Monitoring & Response

Appendix B: Motorola Assessment Instrument

Table B1: Motorola assessment tool.

Score	Approach	Deployment	Results
**Poor (0)**	Management does not recognize the need (OR) Organization does not have commitment (OR) Organization does not have the ability	Organization does not have the practice (OR) Organization does not show interest	Ineffective
**Weak (2)**	Management starts to recognize the need (OR) Support for the practices has recently started (OR) Part of the organization is able to undertake the practice	Usage is scattered (OR) Practice is implemented in some parts of the organization (OR) The practice is only undergoing monitoring/verification	Results are inconsistent (OR) Some effectiveness is shown in some parts of the organization
**Fair (4)**	Wide but not full management commitment (OR) A plan for the practice implementation is defined (OR) Several supporting items are in place	Usage is less fragmented (OR) Practice is implemented in some important parts of the organization (OR) Practice is undergoing monitoring/verification in several parts of the organization	Positive and consistent results in some parts of the organization (OR) Inconsistent results in other parts of the organization
**Marginally qualified (6)**	Some management commitment (OR) Some management members are proactive (OR) Implementation is underway in some parts of the organization (OR) Practices supporting items are in place	Implemented in some parts of the organization (OR) Consistent usage across many parts of the organization (OR) Practice is undergoing monitoring/verification in many parts of the organization	Positive quantifiable results in most parts of the organization (OR) Consistent positive results across many parts of the organization
**Qualified (8)**	Full management commitment (OR) Most management members are proactive (OR) Practice implementation is integrated with the process (OR) Practice improvement is motivated by supporting items	Implemented in almost all parts of the organization (OR) Consistent implementation across almost all parts of the organization (OR) Practice is under monitoring/verification across almost all parts of the organization	Positive quantifiable results in almost all parts of the organization (OR) Consistent positive outcomes across almost all parts of the organization
**Outstanding (10)**	Management has leadership and commitment (OR) Recognition of excellence outside the organization	Consistent implementation across all parts of the organization (OR) Practice implementation is monitored/verified across the organization	Required results are exceeded (OR) World-class results are consistently achieved (OR) Consultancy sought by others

Appendix C: Audit Trail of the Practice Set

Table C1 records every change to the practice set from the 121 candidates produced by Phase 1 to the 117 practices of the final model, with the source of each change and its rationale.

Table C1: Audit trail of changes to the practice set

#	Change	Practice(s) affected	Source	Rationale and outcome
C-1	Merged	Require explicit authorization for every access to cognitive city systems and data, including internal ones.	Expert review (item 5)	Overlapping coverage with CC-BP-40; single consolidated statement retained; coverage unchanged.
C-2	Merged	Verify the identity of all devices, users, and AI agents before connecting to cognitive city networks	Expert review (item 5)	Overlapping coverage with CC-BP-40; single consolidated statement retained; coverage unchanged.
C-3	Merged	Re-verify access for each session instead of trusting previous logins	Expert review (item 5)	Overlapping coverage with CC-BP-40; single consolidated statement retained; coverage unchanged.
C-4	Merged	Treat every cognitive city asset, including internal AI services, model repositories, and data stores, as a resource that requires explicit authorization for each access	Expert review (item 5)	Overlapping coverage with CC-BP-40; single consolidated statement retained; coverage unchanged.
C-5	Merged	Restrict access to AI system internals using principle of least privilege where only authorized personnel and systems can reach sensitive components	Expert review (item 5)	Overlapping coverage with CC-BP-40; single consolidated statement retained; coverage unchanged. Practice count 121 $\rightarrow$ 116.
C-6	Reworded	Develop a cybersecurity investment roadmap prioritizing initiatives based on (ROSI)	Expert review (item 2)	Abbreviation "ROSI" expanded to "Return on Security Investment (ROSI)".
C-7	Reworded	Use identity and access management to control AI training data and decisions	Expert review (item 2)	"Identity and access management" qualified as "role-based and context-aware" to make the assessable control explicit.
C-8	Reworded	Apply dedicated security controls to fog computing nodes before cloud transmission	Expert review (item 2)	Implementation examples added for fog-node controls.
C-9	Reworded	Implement event-driven monitoring with batched telemetry uploads to optimize bandwidth utilization and energy consumption across sensor networks	Expert review (item 2)	Clause added requiring immediate delivery of critical security events
C-10	Added	CC-BP-117 (Monitoring & Response, Level 5)	Expert review (item 4; the expert who rated completeness Neutral)	New practice on autonomous red-team and adversarial testing that continuously evolves attack simulations. Practice count 116 $\rightarrow$ 117.
C-11	No change	—-	Case study (City X)	The City X assessment yielded usability recommendations (color coding, web-based tool) but no change to practice content.
